# Low-grade inflammation may moderate the effect of behavioral treatment for chronic pain in adults

**DOI:** 10.1007/s10865-016-9769-z

**Published:** 2016-07-28

**Authors:** Julie Lasselin, Mike K. Kemani, Marie Kanstrup, Gunnar L. Olsson, John Axelsson, Anna Andreasson, Mats Lekander, Rikard K. Wicksell

**Affiliations:** 1 Psychology Division, Department of Clinical Neuroscience, Karolinska Institutet, Nobels väg 9, 171 65 Solna, Stockholm, Sweden; 2 Stress Research Institute, Stockholm University, Frescati Hagväg 16A, 106 91 Stockholm, Sweden; 3 Institute of Medical Psychology and Behavioral Immunobiology, Universitätsklinikum Essen, Hufelandstr. 55, 45122 Essen, Germany; 4 Behavior Medicine Pain Treatment Service, Karolinska University Hospital, 171 76 Stockholm, Sweden; 5 Department of Physiology and Pharmacology, Karolinska Institutet, 171 77 Solna, Stockholm, Sweden; 6 Osher Center for Integrative Medicine, Karolinska Institutet, Scheeles väg 1, 171 65 Solna, Stockholm, Sweden

**Keywords:** Chronic pain, Cognitive behavioral therapy (CBT), Psychological inflexibility, Chronic low-grade inflammation, Cytokines, Treatment responders

## Abstract

The purpose of the present pilot study was to explore the moderating role of basal inflammation on the effects of behavioral pain treatment in 41 patients with long-standing pain. Baseline pro-inflammatory status moderated behavioral treatment outcomes: higher pre-treatment levels of Tumor Necrosis Factor (TNF)-α and Interleukin (IL)-6 were related to less improvement in pain intensity, psychological inflexibility and in mental health-related quality of life. The treatment outcomes improved in the subgroup that had low levels of pro-inflammatory cytokines at baseline, while the subjects with higher pro-inflammatory status did not. Altogether, results indicate that low-grade inflammation may influence the behavioral treatment outcomes and provide a possible explanation of the heterogeneity in treatment response.

## Introduction

Between 20 and 30 % of the adult population in Europe and in the United States suffer from chronic pain (Breivik et al. 2006; Johannes et al. 2010). Although significant advances have been made in the understanding of chronic pain, a substantial proportion of patients continue to suffer from detrimental effects due to longstanding pain of unclear origin. In addition, pharmacological strategies often fail to reduce symptoms or disability to satisfactory levels (Goldenberg et al. 2004; Turk 2002), which has motivated the development of behavioral interventions aiming at improving self-management of pain. The utility of interventions based on cognitive behavioral therapy (CBT) is supported by a large number of studies (Eccleston et al. 2009; Williams et al. 2012). CBT for pain comprises a diverse array of interventions targeting maladaptive cognitive and behavioral strategies to decrease symptoms and disability. This includes applied relaxation (AR), which constitutes an empirically supported behavioral treatment that focuses on specific relaxation strategies to manage pain (Gustavsson and von Koch 2006; Linton and Gotestam 1984), and Acceptance and Commitment Therapy (ACT), which promotes acceptance of pain and related distress, and engagement in personally valued activities also in the presence of pain (i.e., psychological flexibility) (Trompetter et al. 2014; Wetherell et al. 2011; Wicksell et al. 2013; Williams et al. 2012). However, effect sizes are generally modest and the variability of treatment effects large. Further developments, such as tailoring treatment, by matching interventions with specific subgroups of patients, may improve outcomes but this requires a better understanding of factors that predict or moderate treatment effects in these types of treatment (Eccleston et al. 2009). Unfortunately, despite attempts to identify reliable predictors of treatment effects, characteristics of treatment responders remain unclear.

One potential factor that could modulate the effect of behavioral treatment for chronic pain is inflammation. Alterations in inflammatory processes have been hypothesized as contributor to pain (Watkins and Maier 2005). During acute activation of the immune system, cytokines are known to induce a number of behavioral symptoms, one of which is pain sensitization (Dantzer et al. 2008), and the cytokine interleukin (IL)-8 has been associated with the acute development of hyperalgesia in both rodents and humans (Cunha et al. 1991; Karshikoff et al. 2015). In addition, low-grade increase in systemic concentrations of inflammatory markers, including IL-6, IL-8 and tumor necrosis factor (TNF)-α, have been observed in patients with chronic pain (Gur and Oktayoglu 2008; Kadetoff et al. 2012; Koch et al. 2007; Parkitny et al. 2013; Wang et al. 2008). In sum, these data suggest that inflammatory status could contribute to pain. However, to our knowledge, no study on patients with chronic pain has yet investigated whether inflammatory status before treatment modulates the effect of behavioral treatment.

Hence, the aim of the present pilot study was to explore the influence of inflammatory status on the effects of behavioral treatment in a sample of adults with chronic pain, by assessing the moderating effects of baseline (pre-treatment) inflammation on treatment-induced changes in pain intensity, pain disability and psychological outcomes (i.e., psychological inflexibility and health-related quality of life).

## Methods

The design, sample, assessment, treatment content and effects have been described in detail previously (Kemani et al. 2015), but a brief description is presented below.

### Participants and procedure

Patients were eligible for inclusion if they had longstanding pain (≥6 months), were 18–65 years old, and had a stable pain medication during the past 2 months. Patients were excluded if they participated in a concurrent CBT-based treatment or presented with severe psychiatric co-morbidity that required immediate assessment and/or treatment (e.g., high risk of suicide, psychotic symptoms, and severe depressive episode). The study was approved by the Regional Ethical Review Board in Stockholm, Sweden (Permit Number: 2010/662-31/3), and has been performed in accordance with the ethical standards. Informed consent was obtained from all individual participants included in the study. Sixty patients were included in the study, and 49 initiated treatment.

A randomized controlled design was used to evaluate the effects of the two behavioral treatments (ACT and AR), and an administrator with no involvement in treatment delivery conducted the randomization in blocks of 12 (www.random.org). The ACT and AR interventions followed written protocols and consisted of 12 weekly group sessions (90 min). Both interventions were primarily aimed at improving functioning in the presence of pain. Treatment was delivered by five therapists in the two conditions. In ACT, the psychologists conducted ten sessions and a pain physician with formal training in CBT and ACT conducted two additional sessions (sessions two and eight). In the AR-condition psychologists conducted all 12 sessions. All therapists were licensed clinical psychologists with formal training in CBT, ACT and AR.

**Fig. 1:**
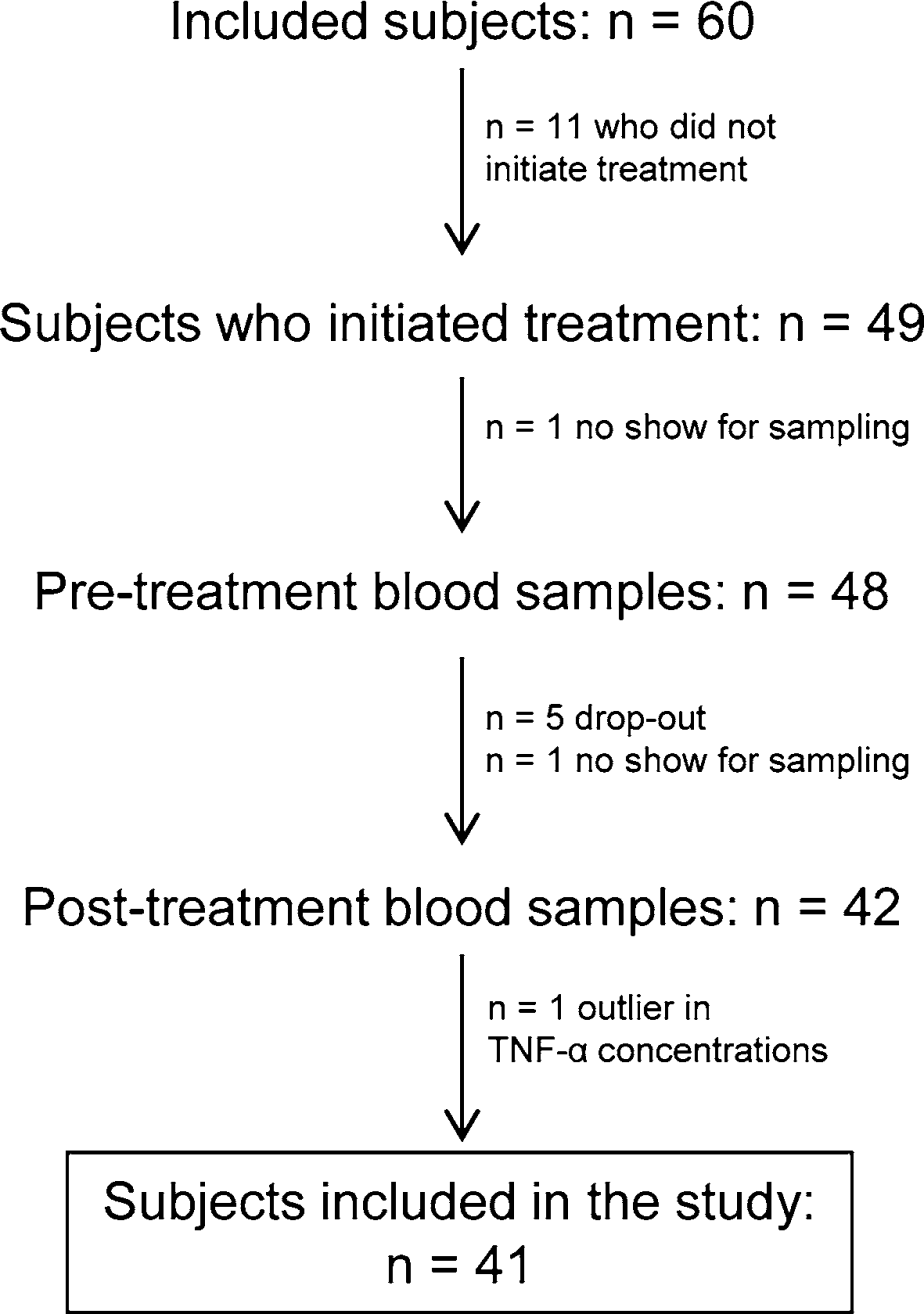
Flow diagram of subjects included in the study

Blood samples were taken at the pre-treatment assessment and immediately after treatment (last treatment session) for all who initiated treatment, except for one who did not show up for sampling before the initiation of treatment. Also, blood samples could not be taken at post-treatment from six other patients (five dropped out from treatment and one failed to show up for post-treatment sampling). In total, blood samples were collected from forty-two patients before and after treatment. One subject who was an outlier (i.e., value higher than mean +4SD) with respect to concentration of TNF-α was excluded. Therefore, forty-one subjects were included in the study (mean age: 40.9 (range 21–61); 32 women; mean duration of pain: 11 years (range 1–36); type of pain: n = 38 (93 %) idiopathic pain, n = 10 (24 %) fibromyalgia, n = 3 (7 %) neuropathic pain; treatment: n = 18 AR, n = 23 ACT) (Fig. 1).

Medication (pain, psychiatric and anti-inflammatory drugs) and self-report psychological measurements (pain intensity, pain disability, psychological inflexibility, health-related quality of life, and mood state; see “Self-report questionnaires” section) were assessed in these patients. One subject did not complete the questionnaire assessing health-related quality of life before treatment.

### Self-report questionnaires

*Pain intensity* during the past week was assessed using a numeric rating scale ranging from 0 (“no pain at all”) to 6 (“extremely painful”).

*Pain disability* was assessed using the Pain Disability Index (PDI), which consists of seven items exploring the range of disability (from 0 to 10—“no trouble” to “total disability”) that the subject experiences in various daily activities (Tait et al. 1987). The reliability and validity of the questionnaire has been analyzed in previous studies, e.g., by Tait (Tait et al. 1990) reporting a Cronbach’s alpha of .86.

*Psychological inflexibility* was measured using the total score of the Psychological Inflexibility in Pain Scale (PIPS) (Wicksell et al. 2008). In ACT, *psychological flexibility* is defined as the ability to notice and accept interfering thoughts, emotions and bodily sensations without acting on them, to facilitate behaving in accordance with personal values and long-term goals also in the presence of those negative experiences. The PIPS comprises 12 items, such as “I cancel planned activities when I am in pain”, rated on a 7-point Likert-scale that ranges from ‘never true’ (1) to ‘always true’ (7). Higher scores indicate greater psychological inflexibility. Results from two previous studies illustrate that the questionnaire has adequate statistical properties (Wicksell et al. 2010).

*Health*-*related quality of life* was assessed using a 12-item short version of the Medical Outcomes Study Short Form (MOS SF)-36 (Ware et al. 1994). Two component scores are derived from the MOS SF-12 scale, the Physical Component Score (PCS) and the Mental Component Score (MCS), which provide measures of physical and mental well-being, respectively. Luo et al. (2003) have reported alphas of .77 (PCS) and .80 (MCS), indicating adequate internal consistency.

*Anxiety and depressive symptoms* were assessed using the total score of the Hospital Anxiety and Depression Scale (HADS) (Zigmond and Snaith 1983). HADS consists of two subscales measuring anxiety (HADS-a), and depression (HADS-d) and has 14 items in total, rated on a 4-point Likert-scale. Results from previous studies show that internal consistency is adequate, .84 for HAD-a and .82 for HAD-d (Lisspers et al. 1997), and reliably measures anxiety and depression in hospital settings (Zigmond and Snaith 1983). Total score of the HADS was used as an index of subjects’ mood state.

### Inflammatory markers

Pre-treatment blood samples were obtained the week prior to treatment initiation and post-treatment blood samples were obtained right after the end of the last session (mean time of blood sample pre-treatment: 11:57; post-treatment: 12:03). Aliquots of serum samples were stored at −80 °C until the assays were performed. Serum concentrations of the pro-inflammatory markers, IL-6, IL-8 and TNF-α, were assayed using high-sensitivity enzyme-linked immunosorbent assay (ELISA) according to the manufacturer’s specifications (R&D Systems, Minneapolis, Minnesota). Sensitivities were 0.039 pg/mL for IL-6, 0.13 pg/mL for IL-8 and 0.106 pg/mL for TNF-α. Intra- and inter-assay variability were respectively ±7.4 and ±7.8 % for IL-6, ±5.5 and ±8.5 % for IL-8 and ±5.3 and ±8.4 % for TNF-α. Concentrations of the three cytokines were log-transformed.

Given that the inflammatory status is probably best defined by a combination of several cytokine concentrations rather than one cytokine only, composite scores of inflammation were calculated using a standard method for identifying latent factors, i.e. Principal Component Analysis, which has been used in several previous studies (Hsu et al. 2009; Lasselin et al. 2012; Lasselin et al. 2014). Two scores were extracted, first using log-transformed IL-6 and TNF-α pre-treatment concentrations (“composite score IL-6/TNF-α”) and then using the three cytokines, IL-6, TNF-α and IL-8 (“composite score IL-6/TNF-α/IL-8”).

### Statistical analyses

The effect of behavioral treatment on self-reported measures (pain intensity, pain disability, psychological inflexibility and health-related quality of life) and inflammatory markers were measured using repeated measures (RM) ANCOVA, adjusting for age, gender, change in the number of used medicines (pain, psychiatric and anti-inflammatory drugs) and change scores of anxiety and depressive symptoms (HADS).

The moderating effect of pre-treatment pro-inflammatory status on the behavioral treatment effects was evaluated using the method of Judd, Kenny and McClelland (2001). According to this method, the change scores (= [post-assessment—pre-assessment]/pre-assessment) in pain-related measures were regressed on each pre-treatment composite score of inflammatory markers, adjusting for age, gender, the changes in the number of used medicines and change scores of anxiety and depressive symptoms.

The effect of behavioral treatment on self-reported measures was then assessed in subjects with higher (i.e., composite score in the highest tertile) versus lower (i.e., composite score in the lowest tertile) pro-inflammatory status before treatment, using RM ANCOVA adjusting for age, gender, change in the number of used medicines and change scores of anxiety and depressive symptoms. Pairwise comparisons (Fisher tests) were used as post hoc analyses.

All statistical analyses were performed using SPSS Statistics 22 (IBM) and a conventional alpha level of 0.05.

## Results

### Effects of behavioral treatment on self-report questionnaires and cytokines

The main effect of behavioral treatment (i.e., across conditions) was not significant on any of the self-reported variables, although tendencies for improvements (*p* = .07–.08) were seen for psychological inflexibility and physical health-related quality of life (Table 1). Behavioral treatment significantly reduced serum TNF-α concentrations (raw values: from 1.74 ± .67 to 1.65 ± .64 pg/mL, RM ANCOVA on log values: F(1,36) = 6.29, *p* = .02, η^2^ = .15) but not IL-6 (raw values: from 1.42 ± 1.39 to 1.39 ± 1.30 pg/mL, RM ANCOVA on log values: F(1,36) = 0.22, *p* = .88, η^2^ = .001) or IL-8 concentrations (raw values: from 13.70 ± 6.04 to 12.62 ± 7.24 pg/mL, RM ANCOVA on log values: F(1,36) = 0.48, *p* = .49, η^2^ = .01).

**Table 1:** Effect of behavioral treatment on pain-related variables

Pain-related variables	Pre-treatment	Post-treatment	*F*	*p*	*η* ^*2*^
Pain intensity	4.3 (0.9)	4.0 (1.4)	0.94	.34	.03
Pain disability (PDI)	39.7 (15.1)	34.2 (16.2)	2.34	.14	.06
Psychological inflexibility (PIPS)	56.4 (10.6)	50.7 (16.7)	3.53	.07	.09
Physical health-related QOL (PCS)^a^	24.5 (11.3)	29.7 (13.4)	3.17	.08	.08
Mental health-related QOL (MCS)^a^	33.7 (9.6)	33.5 (10.8)	0.002	.96	.000

Data shown as mean (SD) across treatments. Repeated measures ANCOVA were performed with age, gender, the change in number of used medicines and the relative change scores of HADS as covariates

*QOL* quality of life, *HADS* Hospital Anxiety and Depression Scale

^a^One subject did not complete this scale

### Moderating effect of baseline inflammation on treatment outcomes

**Fig. 2:**
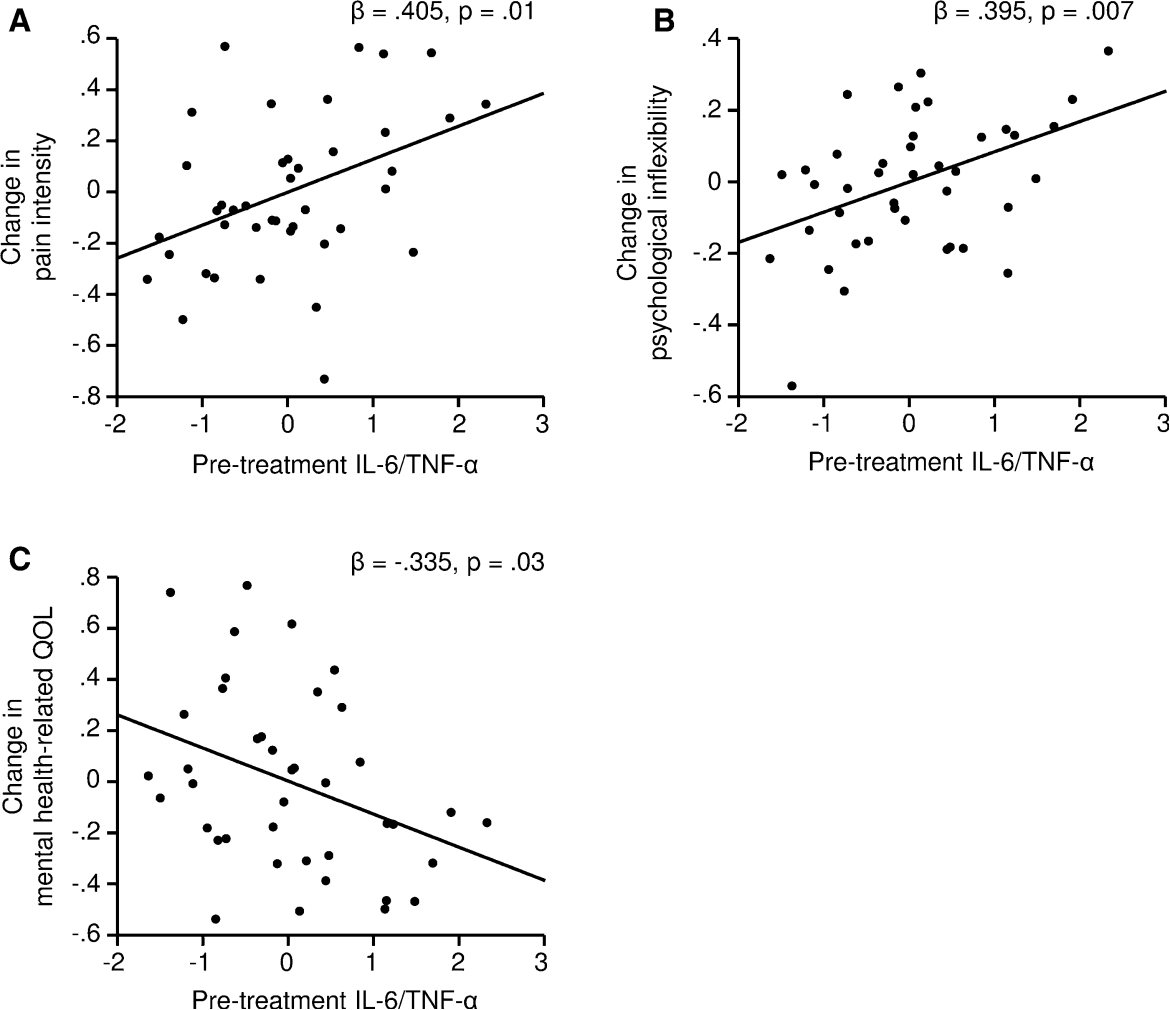
Association between combined concentration of IL-6 and TNF-α before treatment and the change in pain-related variables after treatment. Linear regression analyses, adjusted for age, gender, the change in number of used medicines and the relative change in HADS scores. Changes in pain-related variables were calculated as [= (post-treatment − pre-treatment)/pre-treatment)]. The combined concentration of IL-6 and TNF-α was extracted using principal component analysis

The moderator analyses of pre-treatment inflammatory status on the treatment effect (Table 2) revealed that higher combined concentrations of IL-6 and TNF-α were associated with lesser improvement in pain intensity following treatment. In addition, a significant association between higher composite score of IL-6 and TNF-α concentrations and lower improvement in psychological inflexibility was found. Using the composite score of the three cytokines IL-6, TNF-α and IL-8 reduced these associations. This suggests that higher levels of both IL-6 and TNF-α before treatment were associated with lesser improvement in pain intensity and psychological inflexibility following treatment (Fig. 2a, b). In addition, pre-treatment composite inflammatory scores were negatively associated with the changes in mental health-related quality of life (Fig. 2c).

**Table 2:** Effect of inflammation before treatment on the treatment-induced changes in pain-related variables

Dependent variable	Independent variable	*R* ^*2*^	*β*	*p*
Pain intensity	Composite score IL-6/TNF- α	**.250**	**.405**	**.011**
	Composite score IL-6/TNF- α/IL-8	.190	.308	.053
Pain disability (PDI)	Composite score IL-6/TNF- α	.266	.134	.377
	Composite score IL-6/TNF- α/IL-8	.268	.138	.353
Psychological inflexibility (PIPS)	Composite score IL-6/TNF- α	**.367**	**.395**	**.007**
	Composite score IL-6/TNF- α/IL-8	.294	.275	.064
Physical health-related QOL (PCS)	Composite score IL-6/TNF- α	.077	−.054	.754
	Composite score IL-6/TNF- α/IL-8	.071	−.010	.952
Mental health-related QOL (MCS)	Composite score IL-6/TNF- α	**.311**	**−.335**	**.029**
	Composite score IL-6/TNF- α/IL-8	**.310**	**−.326**	**.030**

Separate linear regression analyses. Dependent variables are the relative changes from pre- to post-treatment [= (post-treatment − pre-treatment)/pre-treatment)]. Independent variables are pre-treatment composite scores, extracted using principal component analyses, using logarithm-transformed IL-6 and TNF-α pre-treatment concentrations (composite score IL-6/TNF-α) or the three cytokines, logarithm-transformed IL-6, TNF-α and IL-8 concentrations (composite score IL-6/TNF-α/IL-8). Regression analyses were adjusted for age, gender, the change in number of used medicines and the relative change in anxiety and depressive symptoms (HADS). Bold font represents significant values (*p* < .05)

*TNF-α* tumor necrosis factor-α, *IL-6* interleukin-6, *IL-8* interleukin-8, *PDI* pain disability, *PIPS* psychological inflexibility, *QOL* quality of life, *PCS* physical health-related quality of life, *MCS* mental health-related quality of life, *HADS* Hospital Anxiety and Depression Scale

**Fig. 3:**
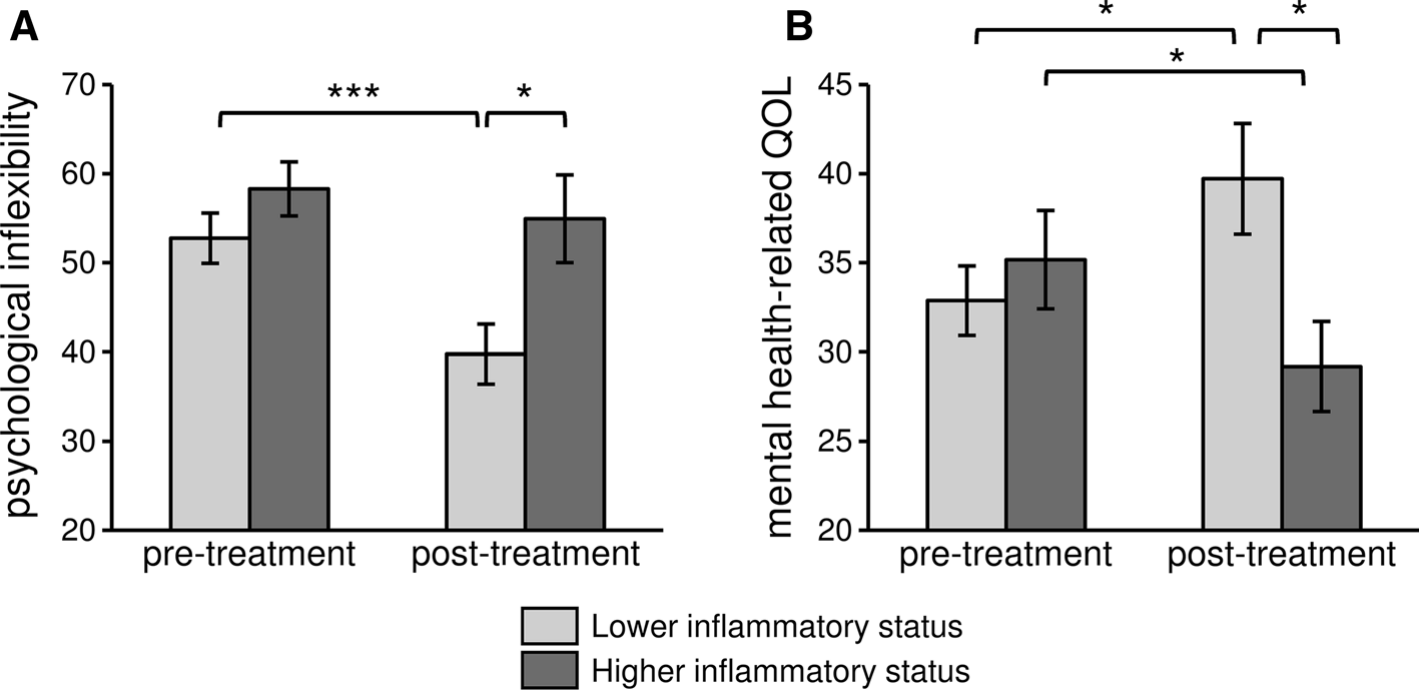
Effect of behavioral treatment in subjects with higher versus lower inflammatory status on psychological inflexibility and mental-health related quality of life. Higher inflammatory status: composite score IL-6/TNF-α/IL-8 in the highest tertile, n = 14; lower inflammatory status: composite score IL-6/TNF-α/IL-8 in the lowest tertile, n = 14. *p < .05; ***p < .001

The effect of behavioral treatment was evaluated in subjects with higher pro-inflammatory status before treatment (i.e., composite score IL-6/TNF-α/IL-8 in the highest tertile; n = 14, n = 9 ACT, n = 5 AR) *versus* lower pro-inflammatory status before treatment (i.e., composite score IL-6/TNF-α/IL-8 in the lowest tertile; n = 14, n = 10 ACT, n = 4 AR) using RM ANCOVA. A significant interaction effect between behavioral treatment and pre-treatment inflammatory status was found for psychological inflexibility and mental health-related quality of life (respectively, F(1, 22) = 4.6, *p* = .04, η^2^ = .17 and F(1, 22) = 10.4, *p* = .004, η^2^ = .32). In particular, behavioral treatment significantly reduced psychological inflexibility (Fig. 3a) and improved mental health-related quality of life (Fig. 3b) only in the group of subjects with lower pro-inflammatory status at baseline.

## Discussion

Results from this pilot study suggest that low-grade inflammatory state can influence the effects of behavioral treatment in patients with longstanding pain. In particular, individuals with higher concentrations of TNF-α and IL-6 before the start of the treatment were more resistant to the improvement in pain intensity and in psychological variables contributing to pain (i.e., psychological inflexibility), as well as in mental health-related quality of life at post-treatment, than patients with lower level of inflammation.

No substantial overall effect of behavioral treatment on pain intensity and pain-related variables was found in the present study. A recent study recently reported that improvements of behavioral outcomes at post-assessment were stronger in ACT than in AR therapy (Kemani et al. 2015), which could explain the absence of significant results when combining the two therapies in the current study. However, the present study aimed at exploring the moderating role of inflammation on behavioral treatment (across conditions). Both therapies were aimed at improving functioning in the presence of pain, and the current findings suggest that pre-treatment inflammatory status may moderate this effect. In particular, results indicate that higher pre-existing levels of IL-6 and TNF-α were associated with reduced effect of behavioral treatment on pain intensity, psychological inflexibility as well as mental health-related quality of life. Additionally, behavioral treatment was found to have an effect on psychological inflexibility and mental health-related quality of life in patients with lower inflammatory status before treatment, i.e., lower combined concentrations of IL-6, TNF-α and IL-8, but not in subjects with higher inflammatory status. Although tentative, the findings from the present study are potentially relevant as they provide a possible explanation of the heterogeneity in treatment response that is commonly seen in patients suffering from chronic pain. Thus, the present study suggests that the inflammatory state may be one of the mechanisms of the persisting behavioral alterations in patients who do not respond to treatment, corresponding to previous studies on treatment resistant depression (Maes et al. 1997; Sluzewska et al. 1997). It also suggests that patients with higher level of inflammation before starting the treatment may benefit from a prolonged therapy or the combination with anti-inflammatory agents. However, these preliminary results should be interpreted with caution and the determination of a precise cut-off associated with non/low response to treatment is not possible from the present data. In addition, the range of inflammatory marker concentrations in patients with chronic pain are usually in the normal range (in the present study, range of IL-6 raw concentrations: .18–5.88 pg/mL; TNF-α raw concentrations: .70–3.36 pg/mL), which implies that inflammatory marker concentration that predict treatment outcomes are not in the pathological range. Further studies are warranted to study and characterize the inflammatory profile that predicts response/non-response to behavioral treatment for chronic pain.

The underlying mechanisms of inflammation in preventing behavioral treatment effects may involve the action of cytokines on the central nervous system (Dantzer et al. 2008). Since most of the effects observed in the present study were stable in relation to mood state, the mechanisms underlying this state of resistance are hypothesized to be independent of an altered inflammation—mood pathway. However, specific psychological factors may be involved. For example, it has been shown that non-responders to pain treatment exhibit poorer coping and higher level of pessimism (Litt and Porto 2013). Interestingly, previous studies have found significant associations of inflammation with higher levels of pessimism and neuroticism in other conditions characterized by a chronic low-grade inflammatory state, i.e., aging and obesity (Capuron et al. 2011; Ikeda et al. 2011; Roy et al. 2010). It seems possible that specific psychological consequences related to a chronic low-grade inflammatory state could prevent the effect of treatment by strengthening the use of maladaptive coping strategies and by reducing the motivational drive.

This study is exploratory in nature and there is a need for further studies on how inflammation may moderate improvements in other populations of patients with chronic pain. The main limitations with the present study were the relatively low number of subjects, which reduced the statistical power, and that the number of analyses led to increased risk for type I-errors. The results should be interpreted with caution and further studies in larger populations are required. Notably, given that some differences in behavioral outcomes were found after the two types of treatment (in favor of ACT in comparison to AR) (Kemani et al. 2015), it would be important to analyze whether the moderating role of inflammation differs across types of behavioral treatment. In addition, comparing patients with and without depression would build on the findings from a recent study showing significant associations between IL-6 concentrations and pain in patients with depressive symptoms, but not in patients without depressive symptoms (Poleshuck et al. 2013). Finally, because of the restricted statistical power, we could not assess the confounding or mediating effect of other factors that may be associated with pre-treatment inflammatory status and therefore could explain the observed relationship between pre-treatment inflammation and treatment outcomes, such as duration of pain or comorbidities. This will need to be disentangled in further studies assessing the mechanisms underlying the effects of inflammation on outcomes of behavioral treatments.

Given that the prevalence of chronic pain is higher in women compared to men (Mansfield et al. 2015), the proportion of women was, as expected, high in the present study (78 %). Although analyses were adjusted for sex, effects may be inflated by an elevated proportion of women. Indeed, results from previous studies suggest that women exhibit higher levels of inflammatory markers and vulnerability to psychological and mood alterations, which are more likely related (Derry et al. 2015). The association of inflammatory status with resistance to behavioral treatment could therefore appear particularly in a female population and the effects in men should be specifically assessed.

A further methodological concern of the present study was the non-standardized timing of blood sampling, due to logistic constraints. Several daily assessments during a number of days, similar to what has been performed in sleep research (e.g., Lasselin et al. 2015), would be impractical but may better reflect factual immune alterations associated with chronic pain and would merit further investigation.

In conclusion, the present study suggests that the pre-treatment inflammatory state may influence the effects of behavioral treatment. The results should be seen as preliminary and further studies evaluating the role of inflammatory cytokines on the success of behavioral treatment of chronic pain conditions are warranted .
